# Cytokine Profiling in Myeloproliferative Neoplasms: Overview on Phenotype Correlation, Outcome Prediction, and Role of Genetic Variants

**DOI:** 10.3390/cells9092136

**Published:** 2020-09-21

**Authors:** Elena Masselli, Giulia Pozzi, Giuliana Gobbi, Stefania Merighi, Stefania Gessi, Marco Vitale, Cecilia Carubbi

**Affiliations:** 1 Department of Medicine and Surgery, Anatomy Unit, University of Parma, Via Gramsci 14, 43126 Parma, Italy; giulia.pozzi@unipr.it (G.P.); giuliana.gobbi@unipr.it (G.G.); cecilia.carubbi@unipr.it (C.C.); 2 University Hospital of Parma, AOU-PR, Via Gramsci 14, 43126 Parma, Italy; 3 Department of Morphology, Surgery and Experimental Medicine, University of Ferrara, 44121 Ferrara, Italy; mhs@unife.it (S.M.); gss@unife.it (S.G.)

**Keywords:** myeloproliferative neoplasms, inflammation, cytokines, megakaryocytes, mutations, polymorphisms, clonal hematopoiesis

## Abstract

Among hematologic malignancies, the classic Philadelphia-negative chronic myeloproliferative neoplasms (MPNs) are considered a model of inflammation-related cancer development. In this context, the use of immune-modulating agents has recently expanded the MPN therapeutic scenario. Cytokines are key mediators of an auto-amplifying, detrimental cross-talk between the MPN clone and the tumor microenvironment represented by immune, stromal, and endothelial cells. This review focuses on recent advances in cytokine-profiling of MPN patients, analyzing different expression patterns among the three main Philadelphia-negative (Ph-negative) MPNs, as well as correlations with disease molecular profile, phenotype, progression, and outcome. The role of the megakaryocytic clone as the main source of cytokines, particularly in myelofibrosis, is also reviewed. Finally, we report emerging intriguing evidence on the contribution of host genetic variants to the chronic pro-inflammatory state that typifies MPNs.

## 1. Overview on Cytokines

Cytokines are soluble factors modulating a plethora of biological processes, including hematopoiesis and immune response. They are low-molecular weight (5–30 kDa) peptides, proteins, or glycoproteins with a very short half-life and active at picomolar concentrations [1].

A structural classification recognize four distinct cytokine categories: (i) the α-helix bundle family (including Interleukin (IL)-2, Interferon (IFN), and IL-10 subfamilies); (ii) the IL-1 family, which primarily includes IL-1 and IL-18; (iii) the IL-17 family; and (iv) the cysteine-knot cytokines, grouping members of the Transforming Growth factor (TGF)-β superfamily. However, as non-structural molecules, their biological properties have been the gold standard for their classification; indeed, the term “cytokine” currently encompasses functional subcategories such as interferons, interleukins, chemokines, growth factors, and adipokines [1].

Cytokine receptors are in turn classified in five major categories: (i) immunoglobulin superfamily; (ii) hematopoietic receptor family; (iii) IFN receptor family; (iv) Tumor Necrosis Factor (TNF) receptor family; and (iv) chemokine receptors [1,2].

Functional redundancy, overlap in biological properties, and pleiotropism are hallmarks of cytokine-mediated effects, and these characteristics represent the main limit for therapeutic interventions aimed to target cytokines and/or their receptors. 

Typically, cytokines are released in response to harmful stimuli, microbial invasion, infected cells, toxic substances, and injury, and act on several cell types to orchestrate the immune response in a tightly regulated balance. Indeed, pro-inflammatory cytokines, promoting the recruitment and activation of immune cells, are normally counterbalanced by anti-inflammatory cytokines, which minimize cellular stress and tissue damage, in a self-limiting process. Deregulated pro-inflammatory cytokine production converts an acute inflammatory response, usually self-limiting and strategically aimed to remove pathogenic noxa and promote healing, into a chronic inflammatory condition, which underlies a number of diseases such as autoimmune and neurodegenerative disorders, atherosclerosis, and cancer [3]. 

## 2. The Concept of Onco-Inflammation

The deep interconnection between chronic inflammation and cancer was effectively encapsulated in Harold Dvorak’s seminal definition of tumors, in the late 1980s, as “wounds that do not heal” [4]. More recently, the concept of “tumor-associated immune dysregulation” has been introduced to describe tumor cell capability to hijack the wound healing program and generate—via pro-angiogenic (i.e., Vascular Endothelial Growth Factor (VEGF), Fibroblast Growth Factor (FGF), and IL-8) and immunosuppressive (primarily IL-10 and TGF-β) cytokines—a permissive microenvironment enabling immunosurveillance escape and tumor growth. At the same time, unresolved chronic inflammation (i.e., chronic infections, autoimmune disorders) can increase the risk of cancer by providing sustained levels of pro-inflammatory cytokines such as TNF-*α*, IL-6, and IL-8, which enhance cell proliferation, inhibit apoptosis, elicit genotoxic stress via reactive oxygen species, favor the epithelial–mesenchymal transition program, and stimulate cell migration. Cancer cells themselves are in turn a relevant source of inflammatory mediators that modify the neighboring stroma, generating therefore a vicious cycle that leads to cancer initiation and progression [5,6]. 

The concept of “onco-inflammation” has been recently proposed to depict this complex cross-talk between cancer cells and their inflammatory microenvironment [7].

However, while in solid tumors the contribution of cytokines, immune cells, and stromal microenvironment in cancer initiation and progression is well-established, in the setting of hematological malignancies, it has for long been elusive [8].

Among different hematological neoplasia, Philadelphia-negative myeloproliferative neoplasms (MPNs) represent the paradigm of onco-inflammatory disorders [9,10].

## 3. MPNs as a Model of Onco-Inflammatory Disorders

The classic Philadelphia-negative chronic myeloproliferative neoplasms (MPNs) are a group of closely-related stem cell disorders, characterized by excessive proliferation and abnormal differentiation of one or more myeloid cell lineages, coupled with varying degrees of bone marrow fibrosis, altered peripheral blood cells count, extramedullary hematopoiesis, and organomegaly [11]. Classical MPN categories include polycythemia vera (PV), essential thrombocythemia (ET), and primary myelofibrosis (PMF), which in turn is divided into a pre-fibrotic (prePMF) and overt stage (overtPMF) [12]. Despite this operational classification, there is a considerable overlap in terms of laboratory findings, symptoms, bone marrow morphology, and genetic profile among these disorders. Moreover, disease mimicry and tendency to phenotypical transformation are common features, and therefore, configure a “biological continuum” among disease entities, with PV and ET representing the early stage and myelofibrosis (MF) the advanced, burnout phase [8,13]. 

Indeed, the natural history of these diseases may predict transformation into post-ET or post-PV myelofibrosis (sMF), and post-MPN acute leukemia (also known as blast-phase MPN, MPN-BP) [14].

The landmark discovery of the *JAK2V617F* mutation ushered a new era in the biology of MPN and established for the first time a link between an acquired genetic hit affecting the neoplastic clone and the major pathway involved in cytokine signaling, i.e., Janus Kinase (JAK)/Signal transducer and activator of transcription (STAT) [15].

The JAK/STAT pathway has a central role in the signaling of cytokines by regulating proliferation, survival, and differentiation of hematopoietic and immune cells. Dysregulation of the JAK/STAT pathway is a hallmark of MPNs as it may result from the occurrence of one of the three so-called “driver mutation”, i.e., *JAK2* exon 14 and exon 12 mutations, thrombopoietin receptor gene (*Myeloproliferative Leukemia Protein, MPL*) mutations, or calreticulin *(CALR)* mutations [16].

In 2005, four research groups independently reported a clonal recurrent somatic mutation located in the pseudokinase domain of *JAK2*, namely, *JAK2V617F*, consisting in a guanine to thymidine point mutation at nucleotide 1849 in exon 14, that results in a valine (Val; V) to phenylalanine (Phe; F) substitution at codon 617 within the JH2 domain of *JAK2*. This substitution is capable to disrupt the autoinhibitory activity of the pseudokinase domain JH2 on the catalytic activity of the kinase domain JH1, leading to a constitutive active tyrosine-kinase [17,18,19,20]

The *JAK2V617F* mutation results therefore in a ‘gain of function’ modification that confers cytokine hypersensitivity and cytokine-independent growth to hematopoietic cells via activation of JAK2-downstream effectors such as STATs, mitogen-activated protein kinase (MAPK), phosphatidylinositol-3′-kinase (PI3K), and AKT/mammalian target of rapamycin (mTOR) [21]. It has been shown that *JAK2V617F* mutation may occur in hemangioblast, a stem cell common to hematopoietic and endothelial cells [22]. Therefore, it is likely that clonal expansion occurs at later stages of differentiation elicited by other somatic mutations (i.e., *TET2, DNMT3A).* These mutations may cooperate with driver mutations to favor clonal dominance and may occur prior to *JAK2V617F*. Indeed, the chronological sequence of mutations affects disease phenotype, particularly for *TET2* and *DNMT3A:* the acquisition of *TET2* or *DNMT3A* prior to *JAK2V617F* mutation leads to an ET phenotype; by contrast, when *JAK2V617F* occurs first, it dictates a PV phenotype. In addition, ‘*TET2*-first’ patients are older at diagnosis while no difference in terms of age of onset is described for *DMT3A* mutation [23,24].

*JAK2V617F* has been reported in more than 70% of MPN, namely, 95% of PV, 50% of ET, and 60% of PMF, and paved the way for the subsequent discovery of *JAK2* exon 12 mutation in 2–3% of PV [25] and, in 2006, of activating mutations in exon 10 of the thrombopoietin receptor gene *MPL,* occurring at the W515 residue [26,27]. When W515 is substituted by 17 other amino acids—mainly leucine (Leu; L) and lysine (Lys; K)—the thrombopoietin receptor (MPL) becomes constitutively active. The mechanism by which these mutations alter MPL signaling relies in the modified geometry of the MPL dimers, leading to transphosphorylation of the pre-bound JAK2 proteins. Therefore, similarly to what happens when a *JAK2V617F* mutation occurs, these events give rise to a constitutively active JAK2/STAT signaling initiated through the thrombopoietin receptor. These mutations are described only in ET and PMF, with frequencies of approximately 3% and 5–8% in ET and PMF, respectively [21].

In 2013, mutations (indel) in the calreticulin gene (*CALR*) were first reported in 25–30% of ET and PMF that were negative for JAK2 and MPL mutations. Calreticulin is a multifunctional protein regulating calcium homeostasis as it binds calcium ions, rendering them inactive. Calreticulin also serves as a chaperone in the endoplasmic reticulum. More than 50 mutations have been described, all of them located in exon 9 and generating a new C-terminal peptide lacking the KDEL sequence required for calreticulin retention in the endoplasmic reticulum [28,29].

While the mechanism by which *JAK2* and *MPL* mutations result in the common activation of the cytokine receptor/JAK2 pathway was straightforward, how *CALR* mutation could unleash hematopoietic stem cell proliferation was far less intuitive. It has been shown that *CALR*-mutated peptides, unlike *CALR*-wild-type that reside in the cytoplasm, are transported to the cellular membrane and activate thrombopoietin receptor [30]. Moreover, *CALR*-mutants acquire a rogue chaperone activity, rescuing the trafficking to the cell surface of defective thrombopoietin receptor mutants that would fail quality check, a function required for the oncogenic effects [31].

In addition to driver mutations, several other genetic hits have been associated with MPN (including *TET2, ASXL1, IDH1/2, EZH2, SRSF2, LNK, CBL*), many of them holding prognostic relevance and variably converging on the activation of JAK/STAT signaling [32], which configures as the common denominator between MPNs and the signaling components of the inflammatory cytokine pathway.

Therefore, current literature agrees that somatic mutations arising in the neoplastic clone (the so-called “bad seed”) can sustain a chronic inflammatory state due to the constant release of inflammatory mediators from in-vivo activated leukocytes and platelets, which alter tissue homeostasis at both a local (bone marrow) and systemic levels. This inflammatory microenvironment, typified by persistently activated immune cells, increased cytokines, accumulation of Reactive Oxygen Species (ROS), tissue damage, remodeling, and fibrosis, represents the so-called “bad soil” which in turn elicits clonal expansion, selection, and evolution [8,13,33,34,35,36].

Embracing a well-established concept in solid oncology, we can observe, in MPNs, the coexistence of the two pathways (intrinsic and extrinsic) that interconnect inflammation and cancer: in the intrinsic pathway, oncogenes drive the generation of an inflammatory microenvironment (in our case, mutant *JAK2*, *CALR*, and *MPL*), while in the extrinsic pathway, chronic non-resolving inflammation increases the risk of neoplasia (in our case, the bone marrow inflammatory milieu).

Key orchestrators at the intersection of the intrinsic and extrinsic pathway include transcription factors (i.e., Nuclear factor-kB (NF-kB); STAT-3), cytokines (i.e., IL-1; TNF; IL-6), and chemokines (i.e., IL-8, Monocyte chemoattractant protein-1 (MCP-1)) [7].

In MPNs, these inflammatory mediators account for systemic symptoms, such as fatigue, weight loss, pruritus, and fever—that are typically exacerbated during the advanced, “burn-out” myelofibrosis stage—and for patient co-morbidities, such as premature atherosclerosis, pro-thrombotic state, and higher risk of second cancer [8,13].

Indeed, an inflammation-driven increase in ROS and Nitric Oxygen Species (NOS) gives rises to epigenetic changes, genomic instability, and additional DNA mutations, which (i) favor the evolution of the original hematopoietic clone from early (i.e., ET, PV, or prePMF) to more advanced stages (i.e., overtPMF, post ET/PV MF) and (ii) can trigger additional genetic events, predisposing to both hematological and non-hematological secondary malignancies [8,13].

In this context, deregulation of the apoptotic process represents a crucial molecular event. The up-regulation of antiapoptotic molecules, such as Bcl-xL and TRAIL (TNF-related apoptosis-inducing ligand), has been documented in MPN. Bcl-xL is a transmembrane mitochondrial protein which inhibits apoptosis by preventing the release of mitochondrial contents such as cytochrome c, while TRAIL conversely induces apoptosis by engaging its receptors (DR4 and DR5) [37,38,39]. Both factors require a fine-tuning during hematopoietic stem cell differentiation [40,41] and their altered expression may lead to aberrant differentiation of hematopoietic progenitors. In fact, Bcl-xL has been found up-regulated in PMF cells [42,43], while a deregulation of TRAIL, DR4, and DR5 expression has been described in ET and PV cells [44], suggesting that alterations in the apoptotic pathways are intrinsic MPN features.

For all these characteristics, Hasselbalch has recently portrayed MPNs as a “human inflammation model for cancer development” in which a chronic inflammatory state is at the core of a self-perpetrating vicious cycle leading to the above-described complications that punctuate MPN natural history [8,10,13].

### Role of the Megakaryocytic Clone in Cytokine Production in MPNs

Aberrant megakaryocytopoiesis is a distinctive feature of MPNs and represents a crucial aspect in MPN pathobiology and differential diagnosis. Megakaryocyte (MK) shape, distribution, and nuclear morphology are a matter of dogmatic debate to distinguish PMF, particularly prePMF (tight clustering, small size, and hypolobulated, “cloud-like” nuclei) from ET (loose clustering, large size, and “staghorn-like” nuclei) [45].

Among the three MPNs, MF is predominantly characterized by profound alterations of megakaryocytopoiesis, such as MK hyper-proliferation coupled with impaired differentiation [42,43,46,47]. As a result, MF patients display alterations in both platelet number (increased or decreased), morphology (presence of giant platelets), and function (reduced ADP-dependent maximal platelet aggregation) [48,49] that may recall inherited platelet disorders [50]. Indeed, as pointed out by Marneth and Mullally, myelofibrosis (intended as fibrosis in the bone marrow) can occur in the context of megakaryocyte disorders, encompassing both inherited bleeding and platelet disorders [51].

By developing a megakaryocyte lineage-specific *JAK2V617F* knock-in mouse, obtained by crossing a conditional *JAK2V617F* knock-in mouse with *Pf4-Cre* transgenic mice, Woods et al. demonstrated that *JAK2*-mutant MK (i) interact with the wild-type hematopoietic milieu to both initiate and sustain MPN; (ii) induce myeloerythroid expansion resulting in a strong PV-like phenotype; and (iii) produce elevated circulating levels of pro-inflammatory cytokines/chemokines, namely, IL-6, C-C motif ligand (CCL)11, C-X-C motif ligand (CXCL)1, and CXCL2 [52].

Indeed, in MF, MK are supposed to be the main source of reactive cytokines that induce fibroblast proliferation, collagen deposition, neoangiogenesis, and osteosclerosis. In particular, MK-derived pro-fibrotic cytokines include TGF-β, Platelet Derived Growth Factor (PDGF), FGF, VEGF, thrombospondin, CXCL4, Macrophage Inflammatory Protein (MIP)-1α, MIP-1β, IL-8, and lipocalin-2 [53]. Among them, functionally-investigated mediators include TGF-β and PDGF/PDGF Receptor (PDGFR) signaling.

TGF-β is a pleiotropic cytokine, with immune-suppressing, anti-inflammatory, and pro-fibrotic properties; it stimulates the production of type I, III, and IV collagen, fibronectin, as well as the synthesis of extracellular matrix components via down-regulation of metalloproteinases and up-regulation of its inhibitors. Additionally, TGF-β promotes cancer invasiveness by favoring endothelial–mesenchymal transition [5]. Studies in a *Gata1^low^*-murine model of MF revealed that extramedullary hematopoiesis is sustained by a circuit between P-selectin and TGF-β, triggered by the abnormal MK expression of P-selectin that leads to neutrophil-megakaryocyte emperipolesis, increasing TGF-β content in the surrounding microenvironment and fibrocyte activation [54,55,56,57].

These results pave the way for a rationale of TGF-β inhibition as a therapeutic strategy in MF [58].

In the same animal model of MF, very recently Kramer et al. investigated the role of PDGFRα- and β-signaling regulation during the development of myelofibrosis in a *Gata1^low^* mouse model. Increased transcript expression of PDGFRα and β as well as the ligand PDGFβ was detected in fibrotic *Gata1^low^* mice; PDGFRβ and PDGFβ ligands are also located in close proximity in overtly fibrotic bone marrow. According to the authors, the most important cell types involved in PDGF signaling are MK, expressing PDGFRα and secreting PDGFβ, and stromal cells expressing PDGFRβ [59].

## 4. Cytokine Profile in MPN Patients

The prominent role of cytokines in the pathophysiology of these neoplasms started to emerge long before the concept of MPNs as a “human inflammation model”, and investigators put extensive efforts to identify a “cytokine signature” that might provide novel pathogenetic insights and serve as a laboratory tool for risk-stratifying patients, predicting and monitoring treatment response. Overall, it can be noted that not all cytokines are repeatedly detected with the same trend in all studies and, in general, some inconsistencies and contradictory findings emerged. This can be due to (i) different methods utilized for cytokine measurement, (ii) the fact that early studies also included chronic myeloid leukemia (CML), (iii) heterogeneous patient populations in terms of disease stage and treatment, and (iv) evolution of MPN diagnostic criteria according to the WHO classification. Moreover, it should be carefully considered that prevalence of MPNs increases with age, and that age is per se a condition associated with a chronic, low-grade, subclinical inflammatory state (the so-called “inflammageing”) [60]. Inflammageing is characterized by elevated levels of circulating inflammatory markers [60], and may therefore represent a bias for cytokine studies in MPNs. Hence, the importance of an age- (and sex-) matched control population is of utmost importance for comparative studies.

### 4.1. Circulating and Bone Marrow Cytokine Levels

In 1999, Hsu and collaborators analyzed the levels of circulating and bone marrow cytokines in N = 70 patients with clonal thrombocytosis caused by myeloproliferative disorders (N = 31 ET, N = 22 PV, and N = 17 chronic myeloid leukemia, CML), N = 28 patients with reactive thrombocytosis, and N = 35 control subjects. The authors observed that both serum and bone marrow Thrombopoietin (TPO), IL-6, soluble (s) IL-6 receptor (R), IL-8, and Stem Cell Factor (SCF) were increased in MPN patients or subjects with reactive thrombocytosis as compared to healthy controls, with IL-6 significantly increased in clonal vs. reactive disorders, and SCF and IL-8 selectively elevated in clonal thrombopoiesis [61]. In the same year, Bourantas and colleagues assessed TNF-α, IL-1α, IL-1β, IL-2, sIL-2R, IL-6, and IL-10 in N = 55 patients with MPNs (N = 10 PV, N = 15 CML, N = 10 PMF, and N = 20 ET) at diagnosis, during follow-up and at the time of blast-phase evolution (which occurred for N = 3 PV and N = 5 PMF), and in 100 control subjects. Surprisingly, serum cytokine concentrations in MPN patients at diagnosis and during the stable phase were either not detectable (IL-lα, IL-1β) or similar to those of normal subjects (IL-6, IL-10), whereas only IL-2 and sIL-2R levels were significantly increased. This elevation was progressive and significantly increased with leukemic evolution [62].

These data are consistent with the findings by Panteli et al., who demonstrated that serum derived from patients with PMF, ET, PV, and CML (N = 25, N = 40, N = 8, and N = 10 patients, respectively) contained significantly higher IL-2 and sIL-2RA than healthy subjects but similar levels of IL-1α and IL-1β. TPO and IL-6 levels were selectively elevated only in PMF. Cytokine levels varied among diseases, with PMF showing increased IL-2, sIL-2RA, IL-6 vs. ET and PV.

Phenotype associations included a negative correlation in all patients between serum IL-2, sIL-2RA, IL-6 with hemoglobin and platelet count, and a positive correlation with spleen and liver size. Analyzing each single disease, only an association between anemia with IL-2 in PMF and sIL-2RA in ET persisted.

Furthermore, in line with the previous work, IL-2, sIL-2RA, and IL-6 levels raised during the transformation phase of CML, during progression of PMF to blast phase, and ET or PV to secondary myelofibrosis [63].

Overall, these initial studies paved the way for the more recent concept of MPNs as a biological continuum with escalation of inflammation as the disease progresses [8,13].

Hermouet and collaborators focused on PV, describing elevated serum levels of IL-8, leptin, Hepatocyte Growth Factor (HGF), and MCP-1 in both PV and secondary erythrocytosis; PV differed from secondary and idiopatic erythrocytosis by lower levels of leptin in serum (male patients only) and higher levels of IL-11 and HGF [64,65]. Gangemi et al. analyzed IL-23, IL-10, and IL-22 in patients with PV and ET showing that only plasma levels of IL-23 were significantly increased in PV but not in ET as compared to controls. No association with history of thrombosis or pruritus was found [66].

Tefferi’s group extensively investigated cytokine expression in MPNs. In their first report in 2007, the authors utilized the human cytokine array method (combining the individual assets of ELISA, enhanced chemiluminescence, and the high-throughput of microspot) in order to detect simultaneously multiple cytokines in the plasma of 20 therapy-naïve MPN patients (N = 10 PMF, N = 5 PV, and N = 5 ET) and N = 4 controls, detecting selectively higher levels of Tissue Inhibitor of Metalloproteinase (TIMP-1), MIP-1, and Insulin-like Growth Factor Binding Factor-2 (IGFBP-2) in PMF patients as compared to ET, PV, and controls [67].

Subsequently, the same group performed a comprehensive analysis of plasma levels by ELISA of 127 PMF patients having complete hematological, histopathological, and cytogenetic information.

As compared to N = 35 healthy controls, PMF patients displayed increased interleukin-1beta (IL-1beta), IL-1RA, IL-2R, IL-6, IL-8, IL-10, IL-12, IL-13, IL-15, TNF-α granulocyte colony-stimulating factor (G-CSF), IFN-α, MIP-1α, MIP-1β, HGF, IFN-γ–inducible protein 10 (IP-10), monokine induced by IFN-gamma (MIG), MCP-1, and VEGF levels as well as decreased IFN-γ levels.

In the N = 90 untreated PMF, increased levels of IL-8, IL-2R, IL-12, IL-15, and IP-10 were independently predictive of inferior survival. At multivariate analysis encompassing the entire study cohort, IL-8, IL-2R, IL-12, and IL-15 prognostic value remained significant after risk stratification, according to the Dynamic International Prognostic Scoring System. Cytokine-phenotype associations included (i) IL-8 with constitutional symptoms, increased leukocytes and peripheral blasts, leukemic evolution; (ii) IL-2R, IL-12, and transfusion need; IP-10 and thrombocytopenia; HGF, MIG, IL-1RA, and marked splenomegaly [68].

As a follow-up of these findings, Pardanani et al. assessed the potential role of increased cytokines in modulating the response to pomalidomide in myelofibrosis (both PMF and post PV/ET MF). They found that elevated circulating levels of MCP-1, IL-2R, IL-15, and IL-8 predicted poor anemia response and that increased levels of sIL-2R, IL-15, and MCP-1 clustered with splenomegaly, which in turn associated with impaired hemoglobin raise during treatment [69].

In 2012, Tefferi’s group expanded previous data by studying the cytokine profile of a cohort of PV (N = 65) compared to the results obtained in their cohorts of PMF (N = 127) and controls (N = 35), finding that PV patients display a unique cytokine profile as compared not only to healthy subjects but also to PMF [70].

As compared to controls, PV presented significantly higher levels of IL-1RA, IL-5, IL-6, IL-7, IL-12, IL-13, IFN-γ, GM-CSF, MIP-1α, MIP-1β, IP-10, MIG, VEGF, HGF, MCP-1, and IL-8 (these last three mediators are in common with Hermouet’s findings [64,65]), while EGF and CCL5 (or RANTES, Regulated on Activation, Normal T cell Expressed, and Secreted) were significantly reduced. Phenotypic correlation in PV included levels of IL-12 with hematocrit; IL-1b, IL-2, IL-7, FGF-β, and HGF with leukocytosis; and IFN-α and IFN-γ with thrombocytosis, MIP-1β with survival in multivariate analysis. As compared to PMF, IL-1β, IL-1RA, IL-2R, EGF, IL-10, FGF-β, IL-12, IFN-α, and RANTES were significantly lower, while IL-7, IFN-γ, GM-CSF, MIP-1α, IP-10, MIG, eotaxin (CCL11), and VEGF were higher.

In 2014, Pourcelot and colleagues reported as well a peculiar cytokine profile according to the MPN subtype, evaluating differences in PV (N = 17) vs. ET patients (N = 21) [71]. As expected, both cohorts displayed elevated cytokine levels as compared to normal values. Cytokines that were differentially expressed in the two groups with significantly higher levels in ET were IL-4, IL-8, GM-CSF, IFN-γ, MCP-1, PDGF, and VEGF. Beyond these cytokines that demonstrated a statistically significant increase, it is worth to underline that the cytokine concentration was globally higher in ET as compared to PV patients, which is quite surprising given that ET is considered, among the three MPNs, the disease with the lowest inflammatory burden [8,13].

In 2018, Cacemiro and co-workers performed an analysis of the different cytokine profiles in all three MPN subsets (N = 11 ET, N = 16 PMF, and N = 20 PV). As compared to controls, elevated plasma levels of the following cytokines—GM-CSF, IFNα, IFNγ, IP-10, MCP-1, MIP-1α, MIP-1β, RANTES, IL-1, IL-4, IL-5, IL-6, IL-10, IL-12, and IL-17—were detected in all three disease categories. Analysis of each disease entity revealed that PMF had higher IFN-γ, IL-12, IL-17, and IP-10 compared to ET and increased IL-12, IL-4, and GM-CSF compared to PV. Cytokine levels were similar in ET and PV, with the exception of RANTES, which was significantly up-modulated in ET [72]. Radar chart representation of the cytokine profile in the three patient cohorts demonstrate that, in accordance with Hasselbalch’s model [8,13], PMF is the MPN subtype characterized by the highest inflammatory burden.

In the same year, the human cytokine array method was utilized by Mambet et al. to investigate plasma cytokine levels in a cohort of young (<35 years) MPN patients (N = 10 PV, N = 10 ET, and N = 10 prePMF): angiopoietin-1, Dickkopf-related protein (Dkk)-1, Epidermal Growth Factor (EGF), IP-9, and PDGF were found elevated as compared to controls, with Dkk-1 levels showing the highest potential for discrimination between MPN subtypes (prePMF > PV > ET) [73].

Barosi et al. focused on sIL2RA in PMF, confirming that PMF patients displayed elevated cytokine levels as compared to healthy controls, and proving that sIL2RA increment correlated with an increased risk of disease progression (indicated by hematological parameters such as decreased hemoglobin and platelet concentrations, increased spleen size, increased percentage of blood blasts, and increased concentration of blood CD34^+^-cells) in *JAK2V617F*-positive but not in *CALR*-mutated patients [74].

Very recently, a joint effort from different research groups allowed to perform a large-scale study of serum cytokine profiles on a considerable cohort of >400 MPNs, leading to the identification of specific inflammatory cytokine signatures according to disease subtypes and evolution. Ten cytokines, namely, INF-γ, IL-1RA, IL-6, IL-8, IP-10, EGF, eotaxin (CCL11), TNF-α, TGF-α, and Growth-Regulated Oncogene (GRO-α) correlated with disease subtypes, disease severity, or overall survival. Specifically, PMF confirmed its association with increased levels of TNF-α, IP-10, and IL-8; ET and PV presented higher eotaxin and EGF, while ET displayed a novel, unique, and specific inflammatory cytokine signature consisting of elevated GRO-α. Surprisingly, hydroxyurea treatment did not affect individual cytokine levels.

In ET, elevated GRO-α at the time of diagnosis correlated also with an increased risk of transformation into secondary MF (but not with blast-phase evolution) and the predictive value of GRO-α remained significant after inclusion of fibrosis grade as a covariate. Of note, the main cellular source of GRO-α in ET has been identified in CD56+/CD14+ monocytes. Authors eventually introduced a novel prognostic model that incorporates cytokine profiling to well-established risk variables such as age, gender, and high-risk mutations [75]. As pointed out by Hasselbalch’s commentary, this study highlights the prominent role of chronic inflammation in modulating MPN phenotype and course, and shows how the measurement of circulating cytokines may be an informative tool for patient risk stratification [76].

### 4.2. Cytokine Gene Expression Profiling

Gene expression profiling in MPNs allowed to identify a number of deregulated genes involved in inflammatory processes and immune response, including cytokines, with the final goal of identifying a distinguished “cytokine gene expression signature” in MPNs.

The results of these studies are not always overlapping with data from circulating/bone marrow measurements, and this may be due to the fact that mRNA levels not always correlate with protein levels. In addition, mRNA transcript assessment does not take into account post-translational modification and sub-cellular/extra-cellular protein localization, affecting protein function.

Additionally, the choice of cellular sources and RNA collection/processing methods is of critical importance. In the MPN setting, the vast majority of studies have been performed on peripheral blood granulocytes [77]. However, this analysis excludes other types of circulating immune cells, such as monocytes, B, and T cells, and blood elements, such as platelets, which are a relevant source of pro-inflammatory mediators. Moreover, microarray studies revealed the presence of transcripts of 1019 different genes in red blood cell RNA of which 107 are genes for immune response [78]. Therefore, whole blood transcriptional profiling can offer a comprehensive analysis of cytokine gene expression panel in these patients.

Indeed, using whole blood transcriptional profiling in a cohort of nine PMF, Skov et al. described a significant up-regulation (top 10 up-regulated genes) of *EGF, HGF*, *CSF3* (encoding for G-CSF), *VEGFA*, and *CXCL9* (encoding for MIG). Indeed, the *HGF* gene was significantly up-regulated across all MPN patients (including also N = 16 ET and N = 36 PV), being most pronounced in PMF, while *VEGFA* was up-regulated in patients with PMF but not in ET and PV. In addition, the authors demonstrated an up-regulation of anti-inflammatory cytokines such as *IL4* (in all three disease categories) and *IL10* (in PV only) [79].

In 2019, Wong et al. [80] analyzed the expression of inflammatory genes in 108 MPNs (N = 13 prePMF, N = 23 overtPMF, N = 31 ET, N = 25 PV, and N = 16 MPN-unclassifiable) by directly measuring RNA transcript abundance in bone marrow biopsies using Nanostring technology, and demonstrated that the inflammatory gene expression profile differs significantly between MPNs with fibrosis grade 0–1 (pre-fibrotic) and MPNs with grade 2–3 (overtly fibrotic). Indeed, pre-fibrotic MPNs display a “low” cytokine gene expression profile, while overtly fibrotic MPNs have marked up-regulation of inflammatory genes. More in detail, chemokine genes such as *CCL2* (encoding for MCP-1) and *CXCL10* (encoding for IP-10) were increase by greater than three-fold; genes encoding for pro-fibrogenic growth factors (*TGFB1, TGFB3*, and *PDGFA*) were upregulated from 2.6- to 1.3-fold; and pro-inflammatory cytokine genes such as *TNF* and *IL1B* were increased by 1.9- and 1.5-fold, respectively. This suggests how distinct inflammatory pathways can lead to a common phenotype of clonal-derived bone marrow fibrosis. On the other hand, it should also be considered that fibrosis itself induces an alteration of bone marrow spatial architecture and cellular composition that may influence inflammatory gene expression profile (up-regulation of genes involved in myofibroblast proliferation, osteoclast differentiation, and angiogenesis) [80].

Table 1, Table 2 and Table 3 summarize the cytokine expression profile in MPNs emerging from the presented studies.

Table 1 shows the cytokine pattern in all three MPNs as compared to healthy subjects. Cytokines are operationally classified according to their function. Overall, all three MPNs displayed an increase of both pro- and anti-inflammatory cytokines, chemokines, growth factors, and fibrogenic mediators, with rare exceptions such as reduction of INF-γ in PMF [68], and of RANTES and EGF in PV [70], not confirmed in other works.

Then, if we compare PMF with ET and PV (Table 2), surprisingly, not all inflammatory mediators are increased. Indeed, of the 29 cytokines whose levels and/or expression have been investigated in PMF as compared to the other two MPNs, 15 are increased (IL-2, sIL-2R, IL-6, IL-12, IL-17, TNF-α, INF-α, IL1RA, IL-4, IL-10, MIP-1β, RANTES, FGF, TPO, TGF-β) [63,70,72,80], IL1-α is similar [63], MCP-1 similar or increased, according to different studies [72,80], five cytokines are decreased (IL-7, VEGF, MIG, GRO-α, and CCL11) [70,75,79], and five show contradictory findings (both increased and decreased according to different reports: INF-γ, MIP-1α, IP-10, GM-CSF, EGF) [70,72,75,80]. The only mediators that are steadily elevated are pro-fibrotic cytokines, such as TGF-β, MCP-1, and FGF (Table 2), suggesting that while inflammation is a common denominator of all three MPNs, PMF is characterized by a unique fibrogenic cytokine signature.

Furthermore, Wong et al. observed an overexpression of fibrogenic genes—particularly of *CCL2*—is not strictly related to overt-PMF per se, but in general to MPNs at overtly fibrotic stage (i.e., MPN-unclassifiable with ≥2 degree of bone marrow fibrosis) [80].

Studies comparing cytokines levels in PV vs. ET are relatively few. As shown in Table 3, a restricted panel of inflammatory cytokines has been tested in ET vs. PV, with substantially no differences in terms of levels of IL-1α, IL-1β, IL-2, sIL-2R, IL-5, IL-6, IL-17, IL-23, IL-10, TNF-α, and INF-α. Surprisingly, the cytokine subtype that revealed primarily elevated in ET is represented by chemokines, growth factors, and fibrogenic cytokines. Specifically, ET shows increased MCP-1, IL-8, RANTES, GRO-α, GM-CSF, PDGF, and VEGF levels as compared to PV (Table 3).

Therefore, based on this literature review, the concept of ET as the form of MPN characterized by a lower inflammatory burden and milder prognosis (lower MF- and AML-transformation rates as compared to PV and survival similar to matched control population [81]) should probably be carefully reconsidered. Indeed, the MPN Landmark Survey clearly demonstrated that ET patients experience a plethora of symptoms that is similar to the other MPN subtype (MF, 78%; PV, 88%; ET, 81%), with fatigue being the most frequently reported [82], and this can be attributable to cytokine deregulation.

## 5. Correlations of Cytokine Profile with Symptoms and Thrombosis

Several studies indicate a correlation between elevated inflammatory mediators and systemic symptoms. Perceived symptoms in MPN patients are highly variable and may include “classic” B-symptoms such as fatigue, fever, pruritus, weight loss, and night sweats, but also anxiety and depression. Specifically, elevated IL-6 levels have been associated to fatigue and depression in a robust cohort of 1788 MPNs [83] and increased IL-8 levels with constitutional symptoms in PMF [68]. IL-4, instead, was correlated with microvascular symptoms in PV patients [70].

Thrombotic events represent the main cause of morbidity and mortality in MPNs [48], and the underlying inflammatory milieu is considered a potential trigger for these complications [13]. Therefore, several groups identified potential pro-thrombotic biomarkers such as high-sensitivity C-reactive protein in PV, ET, PMF, and post-ET/PV MF, as well as pentraxin-3 in PV and ET [34,84,85] and protein kinase C epsilon in PMF and post-ET/PV MF [86]. However, only few studies show a correlation between a history of thrombotic events and the cytokine profile, with often contradictory findings.

Pourcelot et al. described slightly increased levels of IL-12 in the MPN subgroups with vascular complications, but when the analysis was performed for each disease category (PV and ET), surprisingly, IL-12 and GM-CSF were increased in patients without complications [71]. Additionally, in the paper by Vaidya and coworkers, IL-1RA/FGF-β were reduced in PV with arterial thrombosis [70].

Therefore, although dedicated studies are lacking, we can conclude that multiple factors account for the pro-thrombotic state that characterize MPNs.

## 6. Correlations of Cytokine Profile with Driver Mutations

Some authors investigated whether the elevation of specific cytokine(s) could be associated with the presence of phenotypic driver mutation(s), primarily *JAK2V617F*, or other somatic mutation(s) relevant for disease pathogenesis (i.e., *TET2*, *DNMT3A*) and prognosis (i.e., high molecular risk mutations [87]).

Scattered and not univocal associations have been reported, suggesting that somatic mutations (and down-stream activated signaling) in the neoplastic clone are not the only determinant of a MPN inflammatory state.

Pourcelot et al. reported that only TNF-α and PDGF levels were specifically impacted by the *JAK2V617F* status of PV and ET patients [71], while Cacemiro et al. reported the association between the presence of *JAK2V617F* and elevated IP-10 levels in PMF [72]. These data have been recently confirmed in the study by Obro et al., showing a positive correlation of IP-10 with *JAK2V617F* variant allele fraction in ET, PV, and MF patients [75].

In their gene expression analysis, Wong and colleagues observed that *TGFB2* was significantly up-regulated by 3.1-fold in *MPL*-mutated patients [80]. Finally, Barosi et al. demonstrated the prognostic impact of increased sIL2RA levels only in *JAK2V617F*—and not in *CALR*-mutated PMF patients [74].

The variability of correlation among cytokine profiles with driver and (although less investigated) non-driver mutations, is supported by clinical evidences on JAK-inhibitors (primarily ruxolitinib). The effect of JAK-inhibitors relies in their anti-proliferative and anti-inflammatory properties, both concurring, via JAK/STAT inhibition, to a significant reduction of circulating pro-inflammatory cytokines and consequent alleviation of disease-related symptoms. JAK-inhibitors are effective in reducing the inflammatory burden in MPN irrespective of the patient’s *JAK2*-mutational status [88]. Consistently, the efficacy of these drugs on the reduction of *JAK2V617F* allele burden (as surrogate marker of the malignant clone) are controversial. Therefore, a therapeutic strategy based on the combination of different immunomodulatory/anti-inflammatory agents (Interferons, IMIDs, steroids, HDACi) appears rational, potentially providing a more effective disease control [89].

## 7. Host Genetic Variants Predisposing to Chronic Inflammatory State in MPNs

Given this scenario, it is therefore clear that somatic mutations in the neoplastic clone are not the only players in determining the inflammatory burden in MPNs, and other elements, such as inherited genetic predisposition factors, influencing cytokine production, cytokine-signaling activation levels (i.e., NF-kB), and disease phenotype.

The germline *JAK2* 46/1 (GGCC) haplotype consists of a string of four single nucleotide polymorphisms (SNPs) mapping on chromosome 9 and covering a region of about 205 kb, from the *JAK2* intron 10 to the Insulin-like 4 (*INLS4*) gene. These SNPs, namely, rs3780367, rs10974944, rs12343867, and rs1159782, are described in approximately 45% of the general population and their presence strongly predisposes to both *JAK2V617F*- and *JAK2* exon 12-positive MPNs [90], while the association with *MPL*- or *CALR*-mutated disease and with triple negative MPNs remains still controversial [91]. The *JAK2* 46/1 haplotype has also been associated with myelomonocytic phenotype and reduced infection-related survival in normal-karyotype acute myeloid leukemia [92]. Interestingly, this inherited set of SNPs has been linked to non-hematologic chronic inflammatory disorders such as Crohn’s disease [93].

Hermouet et al. [94] envisioned the *JAK2* 46/1 haplotype as a marker of inappropriate myeloid response to cytokines, leading to an enhanced inflammatory state, myeloid neoplasm, and impaired defense against infection. Additionally, the 46/1 haplotype may potentially lead to higher expression of other genes that constitute the haplotype such as *INSL6* and *INSL4*, which in turn may exacerbate pro-inflammatory cytokines production.

Telomerase reverse transcriptase (TERT)—the catalytic subunit of telomerase—is reactivated in up to 90% of all human cancers. The rs2736100 SNP of *TERT*, located in the second intron and accounting for increased enhancer activity, also mediates MPN susceptibility, predominantly in *JAK2V617F*-negative cases [95]. Among non-telomeric function of TERT, a modulation of the expression of IL-6 is described. Since the rs2736100 SNP of *TERT* affects IL-6 expression in solid cancer [96], it has been hypothesized that this inherited variant may represent a potent link between genetic predisposition to cytokine overproduction and MPN onset [9].

Another host genetic variant with a potent link with inflammation is the rs6198 SNP, located in the untranslated region of the glucocorticoid receptor (GR) gene *NR3C1*, whose presence stabilizes the dominant-negative isoform (GRβ) of GR, and therefore interfering with functional isoform GRα [97]. The rs6198 SNP has been variably associated with autoimmune disorders, including impaired glucocorticoid response in rheumatoid arthritis [98]. In MPNs, GRβ expression and the presence of the rs6198 likely contribute to the development of erythrocytosis in PV [99]. In PMF, homozygosity (G/G) for this SNP correlated with a higher white blood cell count, splenomegaly, and elevated circulating CD34^+^ cells. Moreover, in *JAK2V617F*-mutated PMF, the G/G genotype was associated with a shorter overall survival [100].

Our group first described the association of the rs1024611 polymorphism of *CCL2*—encoding for MCP-1—with post-ET/PV MF, and with adverse clinical features in MF (both PMF and secondary MF) patients [101].

Several studies indicate that MCP-1 is increased in all three MPNs (Table 1). By comparing single disease entities, MCP-1 tends to be higher in (i) PMF as compared to ET or PV (Table 2); and (ii) ET as compared to PV (Table 3). MCP-1 is a potent chemoattractant for immune cells in sites of inflammation, promoting multiple pro-inflammatory effects including neoangiogenesis and fibrotic changes [102]. It has been shown that subjects carrying the rs1024611 SNP (originally designated as –2518 A/G or –2578 A/G) produce more chemokine upon inflammatory noxa [103,104]. In a cohort of 177 MPNs (N = 44 PV, N = 65 ET, and N = 68 MF), we found that patients with secondary myelofibrosis are enriched in the polymorphic (G) allele variant and that in overall MF population (both PMF and secondary MF) the SNP was associated with a more severe disease phenotype at presentation (higher IPSS, peripheral blasts, lower hemoglobin, and higher grading of bone marrow fibrosis) [101]. Our data suggested therefore that the rs1024611 SNP of *CCL2* may configure as a genetic biomarker: (i) for the identification of ET and PV patients at higher risk of progression toward a spent phase; (ii) of adverse hematological presentation in MF patients [101].

In addition, it is well-established that MCP-1 signals via NF-kB pathway, a key mediator of onco-inflammation, which in turn promotes the expression of several cytokines, including MCP-1. miR-146a is a negative-regulator of NF-kB pathway whose levels are influenced by the rs24331697 SNP. Ferrer-Marin et al. demonstrated that the rs24331697 T/T genotype, accounting for lower levels of miR-146a, is associated with post-PV/ET MF, shorter MF-free survival in ET and PV patients, and increased plasma inflammatory cytokines in MPNs [105]. These results pave the way for SNP testing in MPNs for risk stratification and outcome perdition. Indeed, as germline variants, there are not affected by clonal evolution or therapy.

## 8. Clonal Hematopoiesis, Cytokines, and Host Genetic Background

Clonal hematopoiesis (CH) is characterized by overrepresented blood cell clones harboring leukemia-associated somatic mutations with a variant allele fraction of >2%. CH is a bona fide pre-malignant condition whose prevalence increases with age and predisposes to several hematologic cancers, including MPNs [106]. CH also confers a higher risk of developing cardiovascular disease via increased cytokine and chemokine expression [107]. Inactivating *TET2* and *DNMT3A* mutations as well as *JAK2V617F* gain of function mutation are the most frequent drivers of clonal hematopoiesis. The loss of function of either *TET2* or *DNMT3A,* although apparently by different patterns of inflammatory gene expression, promotes cardiac hypertrophy, dysfunction, and fibrosis. Indeed, *TET2* inactivation promotes the expression of IL-1β, IL-6, and CCL5, whereas *DNMT3A* inactivation promotes the expression of CXCL1, CXCL2, IL-6, and CCL5 in a lipopolysaccharide-stimulated macrophage cell line [108]. Finally, clonal hematopoiesis arising from the acquisition of *JAK2V617F* mutation can promote cardiovascular disease through pro-inflammatory mechanisms (elevation of IL-6, IL-1β, TNF-α, CCL2) [109].

The influence of heritable factors on CH has been investigated in two recent studies on elderly twins. Fabre et al. performed deep targeted sequencing of blood DNA from 52 monozygotic and 27 dizygotic twin pairs, aged 70–99 years, finding no higher concordance for CH within monozygotic twin pairs as compared to dizygotic [110]. This observation was confirmed in a larger population-based study simultaneously published. The study cohort consisted of 594 twins from 299 pairs, aged 73 to 94 years, and no significant difference in case-wise concordance between monozygotic and dizygotic twins was detected, providing evidence that the inherited genome does not appear to influence the behavior of adult CH [111].

## 9. Conclusions and Perspectives

**Figure 1:**
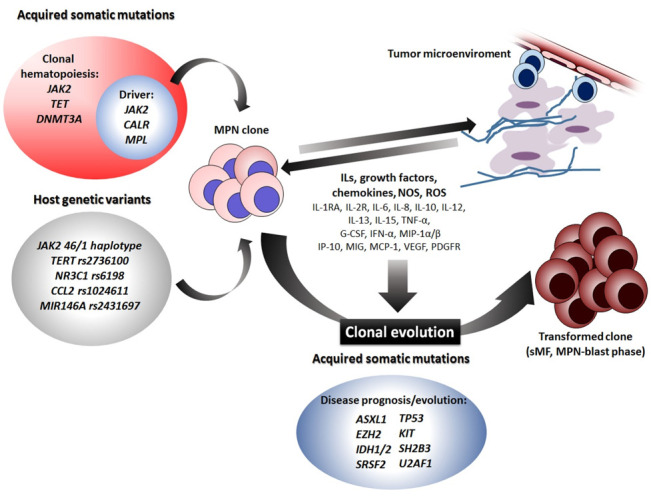
Elements contributing to onco-inflammation in MPNs. The chronic inflammatory state in MPNs is determined by (i) inherited genetic variants of the host; (ii) acquired somatic mutations leading to a premalignant condition (clonal hematopoiesis) first and then to malignant clone expansion; (iii) ILs, growth factors, chemokines, NOS, and ROS released both at a local and a systemic level; (iv) tumor microenvironment (bone marrow immune, stromal, and endothelial cells). All these elements act in turn on the MPN clone eliciting its transformation into a more aggressive disease (sMF or/and blast phase). ILs: interleukins; NOS: Nitric Oxide Species; ROS: Reactive Oxygen Species; sMF: secondary Myelofibrosis.

MPNs represent the paradigm of onco-inflammatory disorders and the malignant clone, primarily the megakaryocytic clone, is at the same time the main source and target of a “cytokine storm” acting both at a local and a systemic level (Figure 1). At a local level, cytokines induce changes to the tumor microenvironment (i.e., the bone marrow) eventually leading to fibrosis, which can be considered as bone marrow end-organ damage. Systemic manifestations include constitutional symptoms, pro-thrombotic state, increased susceptibility to second cancers, and autoimmune disorders. MPN patients can be considered as “dysfunctional cytokine producers”, and, although specific cytokine patterns cannot be outlined, a differential expression of determined cytokines is detected among PV, ET, and MF. This represents also the rationale for the current employment of immune-modulatory drugs such as interferons, IMIDs, and JAK-inhibitors [89]. Despite the JAK/STAT pathway having been traditionally identified as the link between the neoplastic clone and cytokine overproduction in MPNs, correlations among levels of determined cytokines and driver mutations are not univocal. In addition to acquired somatic mutations, inherited host genetic variants, such as SNPs, are emerging as important players in modulating individual pro-inflammatory backgrounds. Therefore, we can envision a scenario in which the acquisition of somatic mutation(s) may result in different MPN phenotypes according to host genetic variations affecting the individual inflammatory state. Hence, the “bad soil” which enables the growth and expansion of “the bad seed” should include constitutional genetic variants.

## Figures and Tables

**Table 1:** Cytokine expression profile in myeloproliferative neoplasm (MPN) subtypes vs. healthy controls.

		ET	PV	PMF	References
Pro-Inflammatory Cytokines	L-1α	=	=	=	[62,63]
	IL-1β	↑/=	↑	↑/=	[62,63,68,72,80]
	IL-2	↑	↑	↑/=	[62,63,68]
	IL-2R	↑	↑	↑	[62,63,68,73,74]
	IL-5	↑/=	↑	=	[68,70,72]
	IL-6	↑/=	↑	↑	[61,62,63,68,70,72]
	sIL-6	↑	nd	nd	[61]
	IL-7	nd	↑	=	[68,70]
	IL-12	↑	↑	↑	[68,70,72]
	IL-13	nd	↑	↑	[68,70]
	IL-15	nd	nd	↑	[68]
	IL-17	=	=	↑/=	[68,72]
	IL-23	=	↑	nd	[66]
	TNF-α	↑/=	↑	↑	[68,72,75,80]
	INF-α	↑	↑	↑	[68,72]
	INF-γ	=	↑	↓/↑	[68,70,72]
Anti-Inflammatory Cytokines	IL-1RA	nd	↑	↑	[68,70]
	IL-4	↑	↑	↑/=	[68,72,79]
	IL-6	↑/=	↑	↑	[61,68,70,72]
	IL-10	↑/=	↑/=	↑	[62,66,68,72,79]
	IL-11	nd	↑	nd	[64,65]
	IL-13	nd	↑	↑	[68,70,72]
Chemokines	MCP-1	↑/=	↑/=	↑/=	[64,65,68,70,72,80]
	MIP-1α	↑	↑	↑	[67,68,70,72]
	MIP-1β	↑	↑	↑/=	[68,70,72]
	IL-8	↑	↑	↑	[61,64,65,68,70,75]
	RANTES	↑	=/↓	↑/=	[68,70,72]
	IP-9	↑	↑	↑	[73]
	IP-10	=	↑	↑	[68,70,72,75,80]
	MIG	nd	↑	↑	[68,70,79]
	GRO-α	↑	=	=	[75]
	CCL11	↑	↑	=	[68,75]
Growth Factors	GM-CSF	↑	↑	↑/=	[70,72]
	G-CSF	nd	nd	↑	[68,79]
	HGF	nd	↑	↑	[64,65,68,70,79]
	PDGF	↑	↑	↑	[73,80]
	VEGF	=	↑/=	↑	[68,70,79]
	EGF	↑	↓/↑	↑	[70,73,75,79]
	FGF	nd	nd	=	[68]
	TPO	=	=	↑	[61,63]
	SCF	↑	nd	nd	[61]
	TGF-β	=	=	↑	[80]
Pro-Fibrotic Cytokines	MCP-1	↑/=	↑/=	↑/=	[64,65,68,70,72,80]
	IL-8	↑	↑	↑	[61,64,65,68,70,75]
	PDGF	↑	↑	↑	[73,80]
	EGF	↑	↓/↑	↑	[70,73,75,79]
	FGF	nd	nd	=	[68]
	TGFβ	=	=	↑	[80]

Summary of deregulated cytokine levels in peripheral blood and bone marrow of essential thrombocythemia (ET), polycythemia vera (PV), and primary myelofibrosis (PMF) patients (by ELISA, cytokine array or by gene expression analysis) as compared to control healthy subjects. Cytokines are grouped according to their function. Cytokines with multiple functions are listed in each category. ↑ increased vs. healthy subjects; = similar to healthy subjects; ↓ reduced vs. healthy subjects; nd: not determined.

**Table 2:** Cytokine expression profile in PMF vs. PV/ET.

		PMF	References
Pro-Inflammatory Cytokines	IL-1α	=	[63]
	IL-1β	↑/=	[70,80]
	IL-2	↑	[63]
	IL-2R	↑	[63,70]
	IL-5	nd	
	IL-6	↑	[63]
	sIL-6	nd	
	IL-7	↓	[70]
	IL-12	↑	[70,72]
	IL-13	nd	
	IL-15	nd	
	IL-17	↑	[72]
	IL-23	nd	
	TNF-α	↑	[72,80]
	INF-α	↑	[70,72]
	INF-γ	↓/↑	[70,72]
Anti-inflammatory Cytokines	IL-1RA	↑	[70]
	IL-4	↑	[72]
	IL-6	nd	
	IL-10	↑	[70]
	IL-11	nd	
	IL-13	nd	
Chemokines	MCP-1	↑/=	[70,72,80]
	MIP-1α	↑/↓	[70]
	MIP-1β	↑	[72]
	IL-8	nd	
	RANTES	↑	[70]
	IP-10	↑/↓	[70,72]
	MIG	↓	[70]
	GRO-α	↓	[75]
	CCL11	↓	[70,75]
Growth Factors	GM-CSF	↑/↓	[70,72]
	G-CSF	nd	
	HGF	nd	
	PDGF	nd	
	VEGF	↓	[70,79]
	EGF	↑/↓	[70,75]
	FGF	↑	[70]
	TPO	↑	[63]
	SCF	nd	
	TGF-β	↑	[80]
Pro-Fibrotic Cytokines	MCP-1	↑/=	[72,80]
	IL-8	nd	
	PDGF	nd	
	EGF	↑/↓	[70,75]
	FGF	↑	[70]
	TGFβ	↑	[80]

Summary of deregulated cytokine levels in peripheral blood and bone marrow of PMF patients (by ELISA, cytokine array or by gene expression analysis) as compared to ET or PV. Cytokines are grouped according to their function. Cytokines with multiple functions are listed in each category; ↑ increased vs. PV/ET; = similar to PV/ET; ↓ reduced vs. PV/ET; nd: not determined.

**Table 3:** Cytokine expression profile in ET vs. PV.

		ET	References
Pro-Inflammatory Cytokines	L-1α	=	[62,63]
	IL-1β	=	[71,72]
	IL-2	=	[63]
	IL-2R	=	[63]
	IL-5	=	[72]
	IL-6	=	[71,72]
	IL-7	nd	
	IL-12	nd	
	IL-13	nd	
	IL-15	nd	
	IL-17	=	[72]
	IL-23	↓	[66]
	TNF-α	=	[71,72]
	INF-α	=	[72]
	INF-γ	↑/=	[71,72]
Anti-Inflammatory Cytokines	IL-1RA	nd	
	IL-4	↑/=	[71,72]
	IL-6	nd	
	IL-10	=	[66,71,72]
	IL-11	nd	
	IL-13	nd	
Chemokines	MCP-1	↑	[71]
	MIP-1α	=	[72]
	MIP-1β	nd	
	IL-8	↑	[71]
	RANTES	↑	[72]
	IP-10	=	[72]
	MIG	nd	
	GRO-α	↑	[75]
	CCL11	=	[75]
Growth Factors	GM-CSF	↑/=	[71,72]
	G-CSF	nd	
	HGF	nd	
	PDGF	↑	[71]
	VEGF	↑	[71]
	EGF	=	[75]
	FGF	nd	
	TPO	=	[63]
	SCF	nd	
	TGF-β	=	[67]
Pro-Fibrotic Cytokines	MCP-1	↑	[71]
	IL-8	↑	[71]
	PDGF	↑	[71]
	EGF	=	[75]
	FGF	nd	
	TGFβ	=	[67]

Summary of deregulated cytokine levels in peripheral blood and bone marrow of ET patients (by ELISA, cytokine array or by gene expression analysis) as compared to PV. Cytokines are grouped according to their function. Cytokines with multiple functions are listed in each category. ↑ increased vs. PV; = similar to PV; nd: not determined.
